# FAT1 knockdown enhances the CSC properties of HNSCC through p-CaMKII-mediated inactivation of the IFN pathway

**DOI:** 10.7150/ijbs.95723

**Published:** 2025-01-01

**Authors:** Jingjing Wang, Yang Chen, Yunqing Sun, Hanzhe Liu, Ruixue Du, Xuewen Wang, Zhe Shao, Ke Liu, Zhengjun Shang

**Affiliations:** 1 State Key Laboratory of Oral & Maxillofacial Reconstruction and Regeneration, Key Laboratory of Oral Biomedicine Ministry of Education, Hubei Key Laboratory of Stomatology, School & Hospital of Stomatology, Wuhan University, China.; 2 Department of Oral and Maxillofacial-Head and Neck Oncology, School & Hospital of Stomatology, Wuhan University, Wuhan 430079, China.; 3 Department of Oral and Maxillofacial Surgery, School & Hospital of Stomatology, Wuhan University, Wuhan 430079, China.; 4 Department of General and Emergency, School and Hospital of Stomatology, Wuhan University, Wuhan, Hubei, China.; 5 Day Surgery Center, School and Hospital of Stomatology, Wuhan University, Wuhan, China.

## Abstract

FAT atypical cadherin 1 (*FAT1*), which encodes an atypical cadherin-coding protein, has a high mutation rate and is commonly regarded as a tumor suppressor gene in head and neck squamous cell carcinoma (HNSCC). Nonetheless, the potential regulatory mechanisms by which FAT1 influences the progression of HNSCC remain unresolved. In this context, we reported that FAT1 was downregulated in tumor tissues/cells compared with normal tissues/cells and that it was correlated with the clinicopathological features and prognosis of HNSCC. Knockdown of FAT1 enhanced cancer stem cell (CSC) properties and decreased the percentage of apoptotic tumor cells. Mechanistically, FAT1 knockdown increased the phosphorylation levels of Ca2+/calmodulin-dependent protein kinase II (CaMKII), subsequently resulting in diminished interaction between phosphorylated STAT1 and interferon regulatory factor 9 (IRF9), which inactivated the interferon pathway and facilitated the adoption of the malignant phenotype of HNSCC cells. The overexpression of STAT1 and IRF9 alleviated the malignant behavior caused by FAT1 inhibition. In summary, our study reveals the role of FAT1 in suppressing the CSC properties of HNSCC via the CaMKII/STAT1/IRF9 pathway, and that targeting *FAT1* might be a promising treatment for HNSCC.

## Introduction

The emergence of cancer is an intricate, multifaceted process intricately linked to both environmental and genetic factors^1^. Mutations in oncogenes and tumor suppressor genes are prevalent across a spectrum of cancers^2^-^4^. FAT atypical cadherin 1 (*FAT1*) has a mutation rate of approximately 22.79% in head and neck squamous cell carcinoma (HNSCC), second only to that of *TP53*^5^-^7^. As a member of the cadherin protein family, FAT1 is involved in a variety of biological processes, including cell proliferation, intercellular communication, and flat cell polarity^7^. Previous investigations have demonstrated that FAT1 deletion strengthens the hybrid epithelial‒mesenchymal transition (EMT) state, leading to the occurrence and metastasis of human squamous cell carcinoma^8^. Nonetheless, the connection between FAT1 and the progression of HNSCC, along with the potential underlying regulatory mechanisms, requires further elucidation.

Interferons (IFNs) are widely known for their role in antitumor therapy and are usually classified into three types: type I (IFNα, IFNβ), type II (IFNγ), and type III (IFNλ)^9^-^11^. In most cell types, cGAS-STING phosphorylates IRF3 after it senses pathogenic DNA, leading to the secretion of type I and type III IFNs^12^, ^13^. Typically, type I and type III IFNs bind to membrane surface receptors, such as IFNAR1/2 (type I IFNs) and IFNLR1 (type III IFNs), and induce the sequential activation of JAKs, TYKs, and STATs to form the transcriptional complex ISGF3, which is composed of p-STAT1/p-STAT2/IRF9^12^-^14^. IFNs exert anticancer effects by decreasing the viability of tumor cells and augmenting the cytotoxic activity of immune cells^15^. Gozgit *et al.* reported that enhanced type I interferon signaling caused by PARP7 inhibition simultaneously inhibits cell proliferation and activates the immune response, resulting in cancer regression^16^. However, the relationship between FAT1 and IFNs has not yet been reported, and whether the type I interferon signaling pathway is involved in FAT1-driven tumor regression still requires clarification.

In this study, we found that the downregulation of FAT1 expression was closely connected with the clinicopathological features of HNSCC. Suppressing FAT1 expression promoted the phosphorylation of CaMKII and restrained the formation of the p-STAT1/IRF9 complex, resulting in deactivation of the interferon pathway and enhancement of the malignant phenotype of HNSCC. Our study revealed the role of FAT1 as a tumor suppressor and underscored its potential as a promising therapeutic target for HNSCC.

## Results

### Downregulated FAT1 expression predicts poor prognoses in HNSCC patients

**Figure 1:**
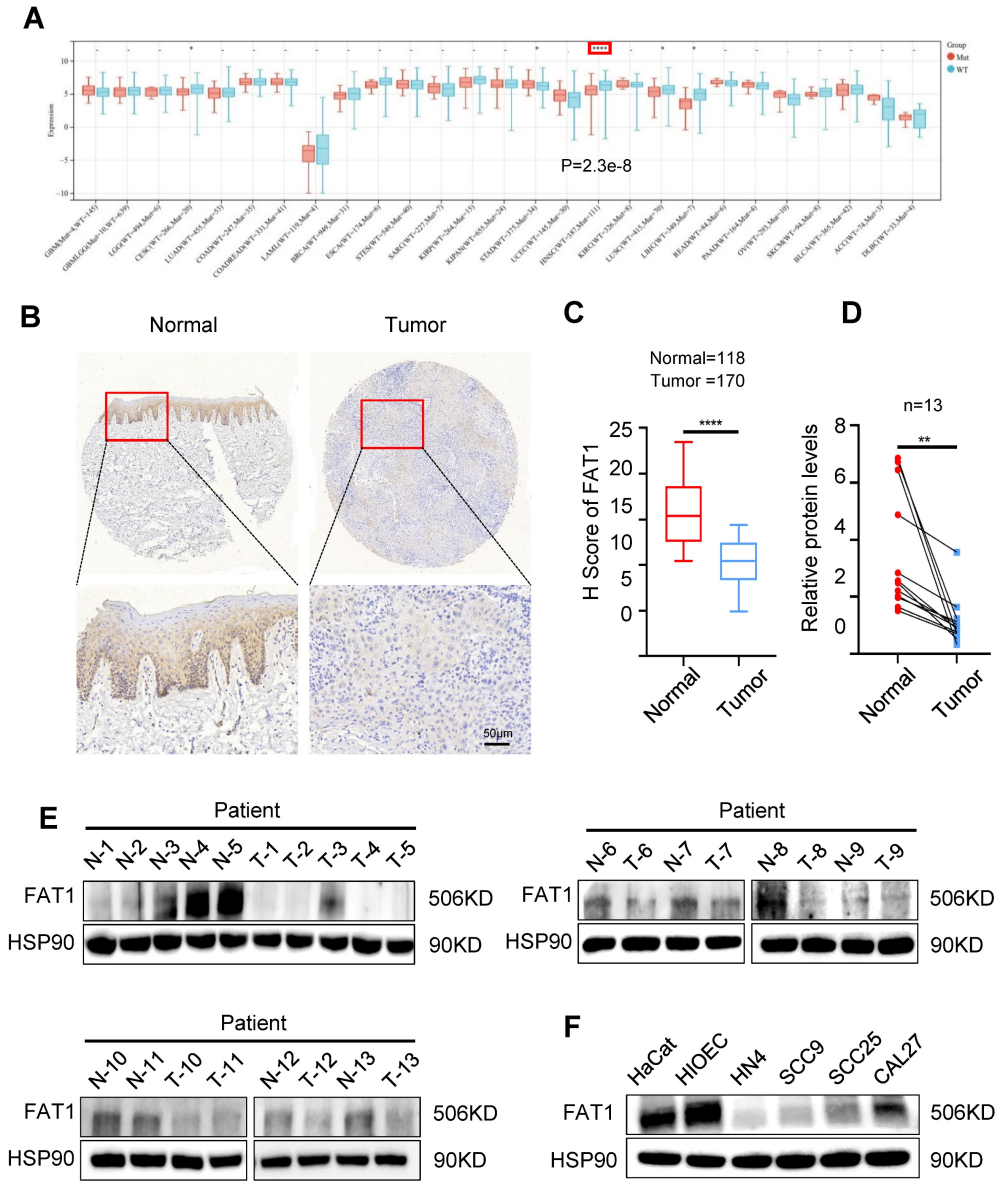
** Downregulated FAT1 predicts poor prognosis of HNSCC. (A)** The expression of FAT1 in HNSCC and normal samples after *FAT1* mutation was acquired from SangerBox.** (B, C)** The expression of FAT1 in HNSCC(n=170) and normal(n=118) tissues., Scale bars, 50 μm. Details information used for statistical purposes are given in Supplementary Excel file 3.** (D, E)** The protein level of FAT1 in HNSCC and normal tissues. HSP90 was used as a control. n=13, Student's t-test. **(F)** The protein level of FAT1 in HNSCC and HIOEC, HaCat. Independent experiments (*in vitro*) were performed in triplicate. Data are presented as mean±SD. *, P < 0.05, **, P < 0.01, ***, P < 0.001, ****, P < 0.0001.

The Cancer Genome Atlas (TCGA; https://www.cancer.gov/ccg/) database revealed that* FAT1* is one of the most frequently mutated genes in HNSCC, second only to *TP53*, and its mutation rate is 22.79% (Fig. S1A). The analysis results acquired from the online tool SangerBox indicated that the expression of FAT1 was significantly decreased following mutation in HNSCC (Fig. 1A). In addition, FAT1 was negatively correlated with distant metastasis but not cervical lymph node metastasis, stage, grade, age and sex. (Fig. S1B-G). We subsequently detected the expression of FAT1 in clinical HNSCC samples and found that the expression of FAT1 was significantly lower in tumor tissues than in normal epithelial tissues (Fig. 1B-E). Furthermore, we examined the expression of FAT1 in the normal epithelial cell lines HIOEC and HaCaT and the HNSCC cell lines HN4, SCC9, SCC25, and CAL27 and found that FAT1 was more highly expressed in normal epithelial cell lines than in squamous carcinoma cell lines. Among the four HNSCC cell lines, FAT1 levels remained at a relatively higher level in CAL27 and SCC25 cells (Fig. 1F). In conclusion, FAT1 expression was downregulated upon mutation in HNSCC, and its expression was negatively correlated with the poor prognosis of HNSCC patients, suggesting a tumor-suppressive role in HNSCC.

### Knockdown of FAT1 enhances CSC properties

**Figure 2:**
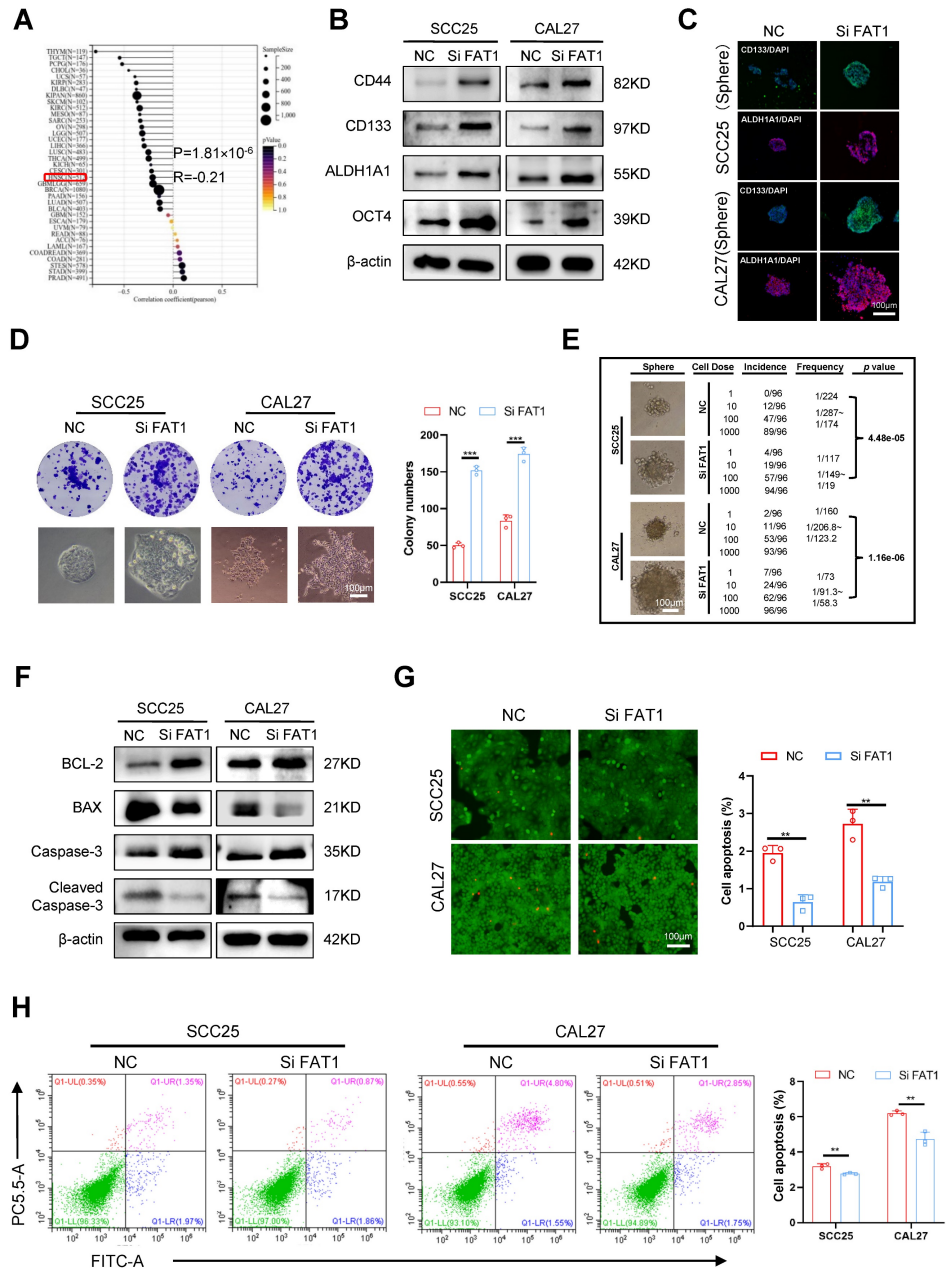
** Knockdown of FAT1 enhances the CSCs properties. (A)** The relationship between the expression of FAT1 and tumor cell stemness was acquired from SangerBox, details information used for statistical purposes are given in Supplementary Excel file 6. R=-0.21, P=1.81×10^-6^. **(B)** Western blot results of CSC-related markers in CAL27 and SCC25 cells after FAT1 knockdown. β-actin was used as a control. **(C)** Cell immunofluorescence of CD133 and ALDH1A1 expression in FAT1 knockdown sphere cells. Scale bars, 100 μm.** (D)** Colony formation assay was performed to examine the colony formation ability of FAT1 knockdown cells. Student's t-test. Scale bars, 100 μm. **(E)** Four doses (1000, 100, 10, and 1) of FAT1 knockdown CAL27 and SCC25 cells were used for limit dilution assay. Frequency was determined by the ELDA web tool. n = 96. **(F)** Western blot results of apoptosis-related markers in CAL27 and SCC25 cells after FAT1 knockdown. **(G)** Live/dead viability assay was conducted to examine the apoptosis rate after FAT1 knockdown. Scale bars, 100 μm.** (H)** Apoptosis experiments were conducted to examine apoptosis rate after FAT1 knockdown. The X axis (FITC) stands for annexin, and the Y axis (PC5.5-A) for PI. Student's t-test. Independent experiments (*in vitro*) were performed in triplicate. Data are presented as mean±SD. *, P < 0.05, **, P < 0.01, ***, P < 0.001, ****, P < 0.0001.

Given that downregulated FAT1 expression is associated with a poor prognosis in HNSCC patients, we wondered whether FAT1 could regulate the malignant behaviors of tumor cells. Through the results acquired from SangerBox, we found that FAT1 expression was negatively correlated with the CSC properties of HNSCC (Fig. 2A). Considering that the expression of FAT1 in SCC25 and CAL27 cells was greater than that in the other HNSCC cell lines (Fig. 1F), we used three different siRNAs to interfere with FAT1 expression in CAL27 and SCC25 cells and selected the most effective sequence for the following experiments (Fig. S2A). The levels of the CSC-related markers CD133, CD44, ALDH1A1, and OCT4 were increased at both the protein and RNA levels after FAT1 knockdown (Fig. 2B and Fig. S2B). In addition, inhibition of FAT1 expression enhanced the sphere formation and colony formation abilities of tumor cells (Fig. 2D and Fig. S2C), and CSC-related markers were robustly expressed in the formed spheres (Fig. 2C). A limiting dilution assay is commonly used to detect the frequency of tumor initiation in CSCs^17^. Here, we found that FAT1 knockdown markedly increased the stemness of tumor cells (Fig. 2E) and increased their proliferative capacity (Fig. S2D, E). In addition, the migration and invasion abilities of cancer cells were enhanced after FAT1 knockdown (Fig. S2F-I). Tumor cells with stronger CSC properties are resistant to apoptosis^18^, ^19^. Western blot and RT‒qPCR results revealed that BCL-2 and Caspase3 were upregulated, whereas Bax and cleaved Caspase3 were downregulated in FAT1-knockdown cells (Fig. 2F and Fig. S2J). Moreover, the FAT1 knockdown group had fewer dead cells, indicating that FAT1 inhibition enhanced the antiapoptotic ability of tumor cells (green signifies living cells, whereas red denotes dead cells; Fig. 2G, H). In summary, these results demonstrated that FAT1 knockdown enhanced the CSC properties in HNSCC.

### FAT1 regulates the type I interferon pathway independent of IFN secretion

**Figure 3:**
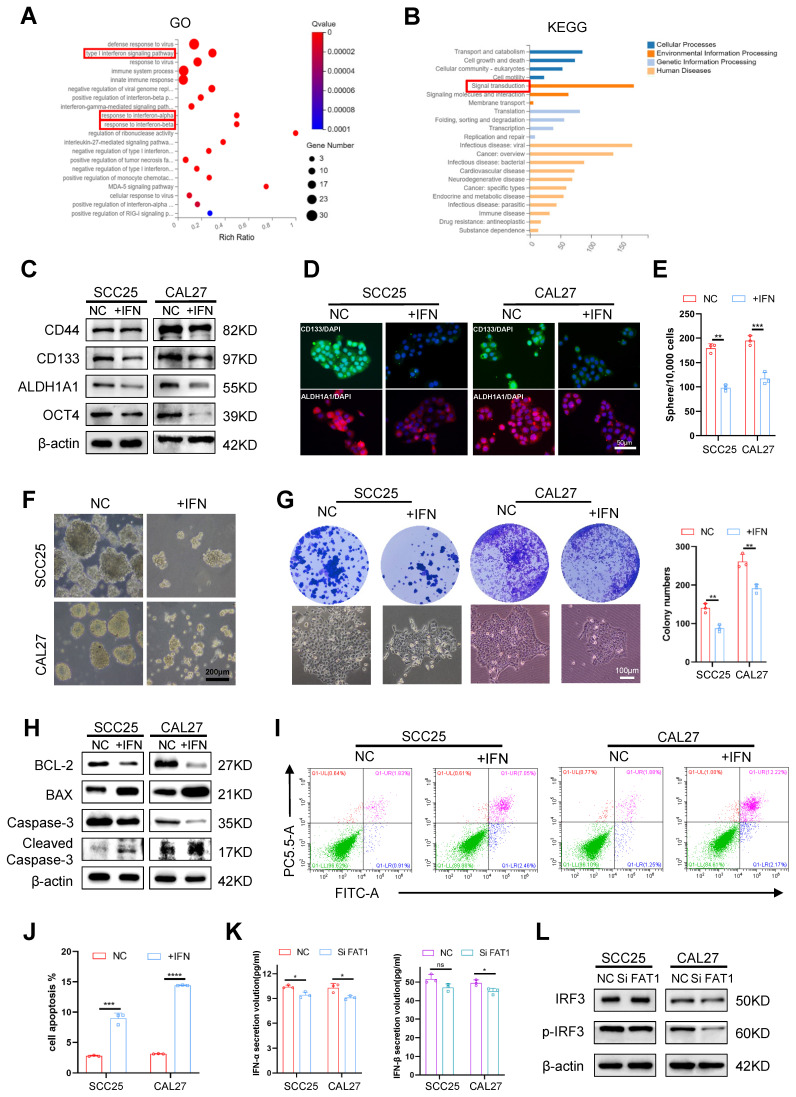
** FAT1 regulates type Ⅰ interferon pathway independent of IFNs secretion. (A)** Gene ontology (GO) analysis obtained from RNA-seq with FAT1 knockdown. **(B)** Kyoto Encyclopedia of Genes and Genomes (KEGG) obtained from RNA-seq with FAT1 knockdown. **(C)** Western blot results of CSC-related markers in CAL27 and SCC25 cells following exposure to the IFN-α for up to 24 h at 100ng/ml concentration. β-actin was used as a control. **(D)** Cell immunofluorescence of CD133 and ALDH1A1 expression in cells exposed to the IFN-α for up to 24 h at 100ng/ml concentration. Scale bars, 50 μm.** (E, F)** Sphere formation assay was used to examine the sphere forming ability of cells treated with IFN-α at 100ng/ml concentration for 24 h. Scale bars, 200 μm. Student's t-test. **(G)** Colony formation assay was performed to examine the colony formation ability of HNSCC cells treated with IFN-α at 100ng/ml concentration for 24 h. Student's t-test. Scale bars, 100 μm.** (H)** Western blot results of apoptosis-related markers in cells treated with IFN-α at 100ng/ml concentration for 24 h. **(I, J)** Apoptosis experiments were conducted to examine apoptosis after being treated with IFN-α at 100ng/ml concentration for 24 h. The X axis (FITC) stands for annexin, and the Y axis (PC5.5-A) for PI. Student's t-test. **(K)** IFN-α and IFN-β concentrations in the supernatants of HNSCC cells after FAT1 knockdown were determined by ELISA. Student's t-test. **(L)** Western blot results of IRF3 and p-IRF3 in CAL27 and SCC25 cells following FAT1 knockdown. `Independent experiments (*in vitro*) performed in triplicate. Data are presented as mean±SD. *, *P* < 0.05, **, *P* < 0.01, ***, *P* < 0.001, ****, *P* < 0.0001.

Previous results have shown that FAT1 deletion enhances the CSC properties in HNSCC. To further explore the underlying mechanisms by which FAT1 regulates the CSC phenotype, RNA sequencing was performed on three biological replicates from each of the SiFAT1 groups and each normal group. The cutoff used to determine DEGs was logFC=0.263 (Q-value=0.05). A total of 268 upregulated and 281 downregulated differentially expressed genes (DEGs) were identified, and a volcano plot was constructed to provide a clearer visual representation of the DEGs (Fig. S3A). Kyoto Encyclopedia of Genes and Genomes (KEGG) and Gene Ontology (GO) analyses revealed that FAT1 knockdown markedly influenced signal transduction, which is closely related to the type I interferon signaling pathway (Fig. 3A, B). Growing evidence has demonstrated that the interferon signaling pathway can directly inhibit tumor growth^1^-^3^. Stimulation by exogenous IFNs leads to the activation of the interferon pathway^20^, ^21^. Therefore, exogenous IFN-α was used to treat HNSCC cells in our research. Western blot, RT‒qPCR and immunofluorescence results revealed that the expression of CSC-related markers clearly decreased in CAL27 and SCC25 cells after treatment with exogenous IFN-α (Fig. 3C, D and Fig. S3B). In addition, the self-renewal and proliferation abilities of the treated group were diminished (Fig. 3E-G). Correspondingly, BCL-2 and Caspase3 were downregulated, and Bax and cleaved Caspase3 were upregulated in cells exposed to IFN-α (Fig. 3H and Fig. S3C). The number of dead cells also increased robustly in the IFN-α-treated group (Fig. 3I, J and Fig. S3D, E). The above results demonstrated that activation of the interferon pathway inhibited the CSC properties of HNSCC. Therefore, we hypothesized that FAT1 knockdown might inactivate the type I interferon signaling pathway by blocking the secretion of type I IFNs. However, we found that the addition of exogenous interferon to tumor cells did not affect the protein expression level of FAT1(Fig. S3F). Besides, the results confirmed that FAT1 inhibition had only a slight effect on the secretion of IFN-α and IFN-β (Figure 3K). The expression of IRF3, a critical protein for type I IFN production, was nearly unchanged (Figure 3L). To demonstrate that IFN-α and IFN-β are not involved in the regulation of the type I IFN pathway by FAT1 knockdown, we interfered with the expression of IFN-α or IFN-β via siRNA treatment while knocking down FAT1 (Fig. S3G, H). Our findings demonstrated that interfering with IFN-α and IFN-β had a negligible effect on FAT1-mediated alterations in the interferon pathway (Fig. S3I), suggesting that FAT1 regulates the interferon pathway in a nonclassical manner independent of IFN secretion. Overall, the above results indicate that FAT1 regulates the interferon pathway through a noncanonical pathway independent of IFN secretion, thereby controlling the CSC properties in HNSCC.

### FAT1 activates the type I interferon pathway through CaMKII dephosphorylation

**Figure 4:**
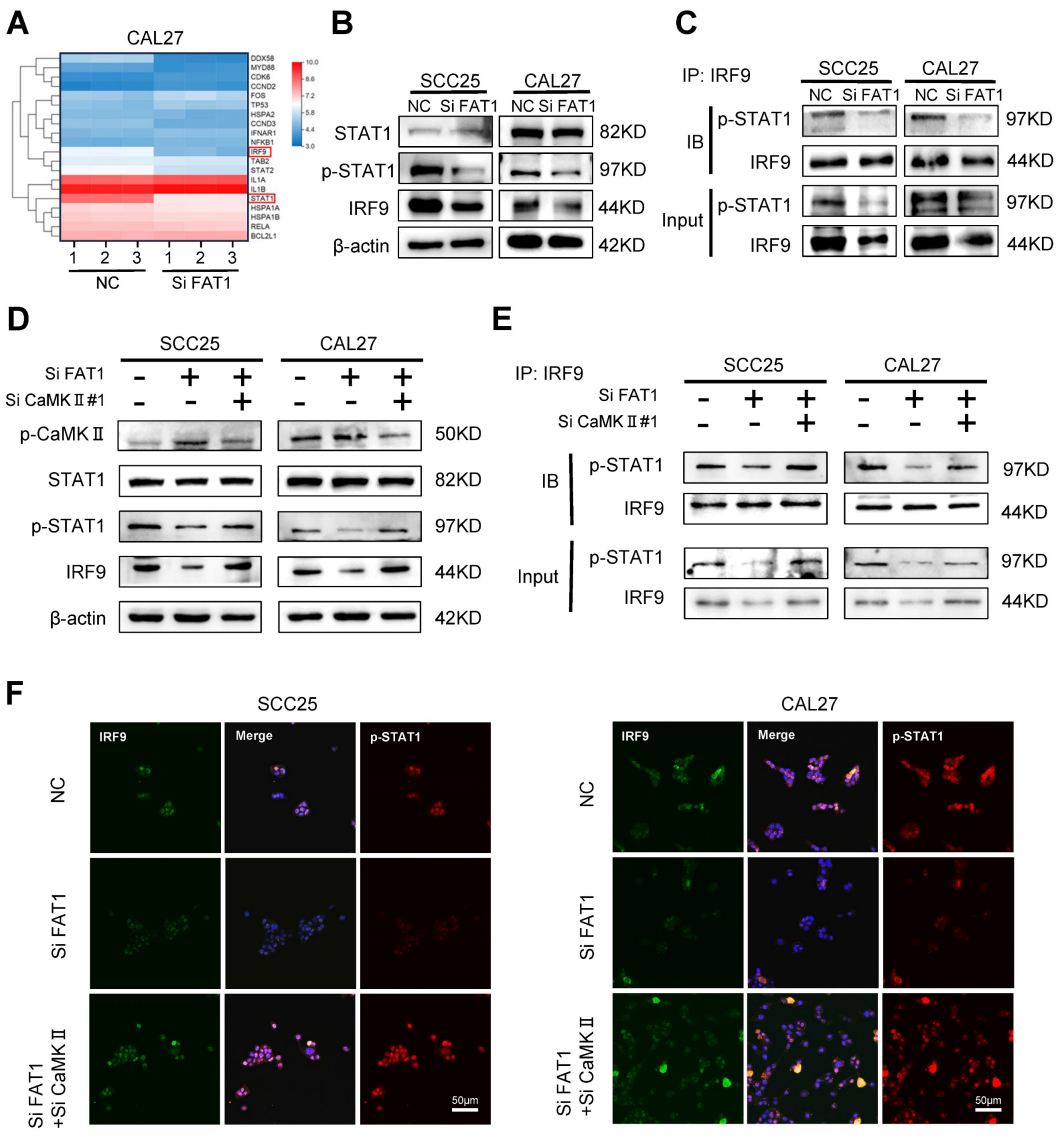
** FAT1 activates type Ⅰ interferon pathway through CaMKII dephosphorylation. (A)** Heat map of the top 20 DEGs identified in CAL27 cells after FAT1 knockdown. Details information used for statistical purposes are given in Supplementary Excel file 4.** (B)** Western blot results of STAT1 and p-STAT1 in CAL27 and SCC25 cells after FAT1 knockdown. β-actin was used as a control. **(C)** HNSCC cells transfected with SiFAT1 or control siRNAs were lysed for 0.5 h on the ice. Lysates were processed for immunoprecipitation (IP) with a control IgG (IgG) or anti-IRF9 antibody. Western blot was used to detect p-STAT1 co-precipitated with IRF9. **(D)** Western blotting results of STAT1, p-STAT1, p- CaMKII and IRF9 after knockdown of FAT1 in tumor cells while knocking down CaMKII. **(E)** HNSCC cells transfected with Si FAT1 were lysed for 0.5 h on the ice while knocking down CaMKII. Lysates were processed for immunoprecipitation (IP) with a control IgG or anti-IRF9 antibody. Western blot was used to detect p-STAT1 co-precipitated with IRF9. **(F)** Immunofluorescence was used to visualize the p-STAT1/IRF9 complex in concomitant FAT1 knockdown cells knocked down for CaMKII. Scale bars, 50 μm. Independent experiments (*in vitro*) were performed in triplicate. Data are presented as mean±SD. *, *P* < 0.05, **, *P* < 0.01, ***, *P* < 0.001, ****, *P* < 0.0001.

RNA sequencing results showed that among the top 20 differentially expressed genes (DEGs), STAT1 and IRF9, which are closely related to the type I interferon signaling pathway, were significantly down-regulated after the knockdown of FAT1 (Fig. 4A). Next, we selected 8 DEGs from the top 20 most differentially expressed for protein‒protein interaction network (PPI) analysis and found that STAT1 was closely related to IRF9 (Fig. S4A). In accordance with the RNA sequencing results, western blot analysis revealed that inhibition of FAT1 markedly reduced the expression of IRF9 and the phosphorylation of STAT1. However, the other component of the ISGF3 complex, p-STAT2, did not significantly change (Fig. 4B and Fig. S4B). Next, equal amounts of magnetic beads were used to conjugate the IRF9 antibody to ensure consistent levels of IRF9 between different groups. The IRF9 antibody-conjugated beads were used to pull down p-STAT1, and we found that FAT1 knockdown directly inhibited the formation of the p-STAT1/IRF9 complex (Fig. 4C and Fig. S4C). Previous studies have shown that CaMKII is the kinase that acts downstream of FAT1^4^. Therefore, we explored whether FAT1 could regulate the activity of CaMKII and found that FAT1 knockdown resulted in the phosphorylation of CaMKII. In addition, we found that the increased phosphorylation level of CaMKII led to the inhibition of STAT1 phosphorylation. We then interfered with the expression of CAMKII via treatment with siRNAs while knocking down FAT1 and found that the expression level of p-STAT1 was restored (Figure 4D and Figure S4D). The coimmunoprecipitation (Co-IP) and fluorescence colocalization results confirmed that p-CaMKII could inhibit the formation of the p-STAT1/IRF9 complex by competitively inhibiting the phosphorylation of STAT1 (Figure 4E, F and Fig. S4E). Taken together, these results demonstrate that FAT1 knockdown blocked the formation of the p-STAT1/IRF9 complex by phosphorylation of CaMKII.

### The overexpression of STAT1 and IRF9 alleviated FAT1 knockdown-driven CSC properties

**Figure 5:**
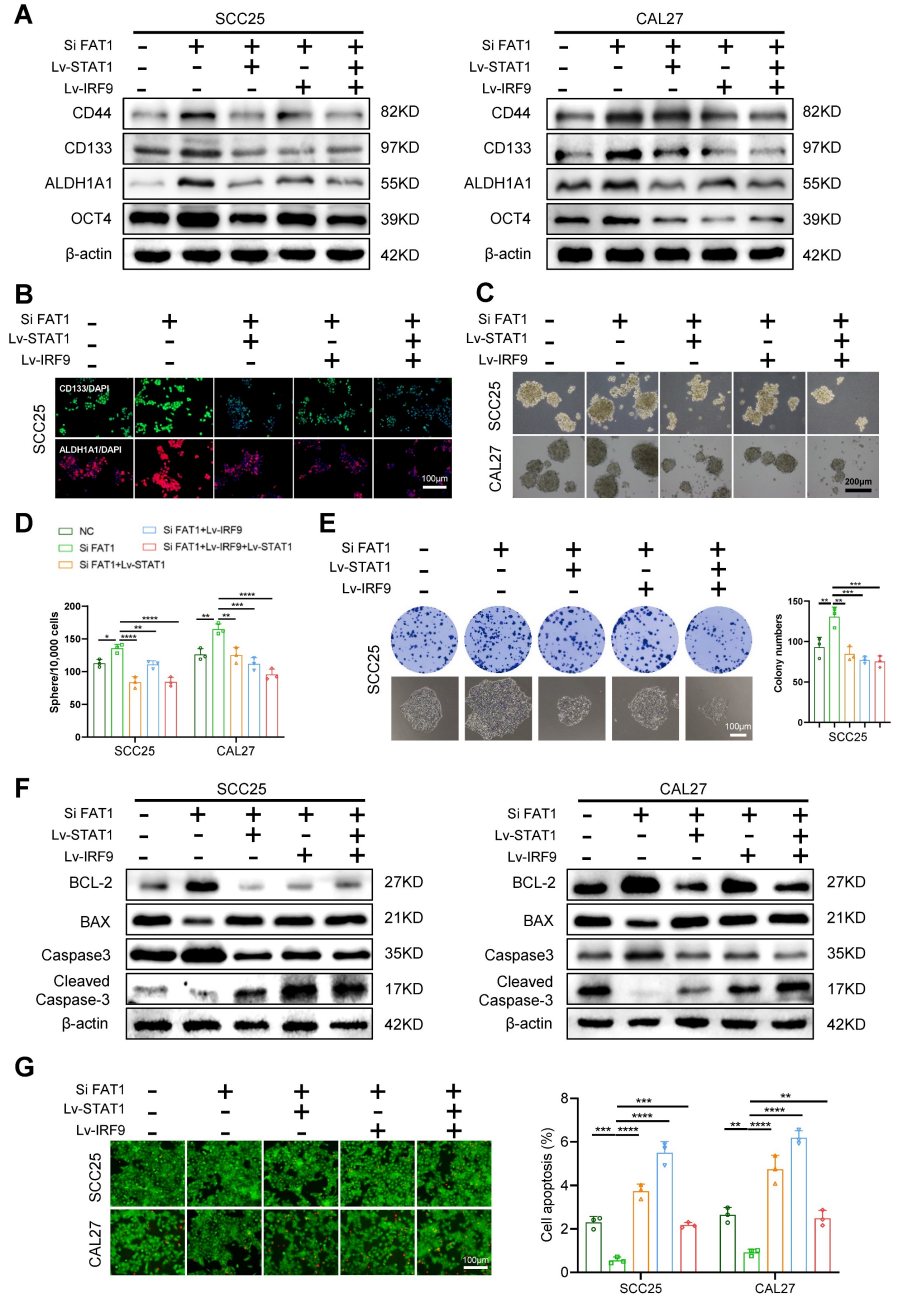
** Overexpression of STAT1 and IRF9 alleviates FAT1 knockdown-driven CSC properties. (A)** CAL27 and SCC25 cells co-transfected with the empty carrier, Lv-STAT1, Lv-IRF9 or Si FAT1 were divided into five groups, and then the protein levels of CSCs-related markers were examined by western blot. β-actin was used as a control. **(B)** Five groups were described in Figure 5A, and the expressions of CD133 and ALDH1A1 in SCC25 cells were determined by cell immunofluorescence. Scale bars, 100 μm. One-way ANOVA. **(C, D)** Sphere formation assay was used to examine the sphere forming ability of cells in five groups. Scale bars, 200 μm. One-way ANOVA. **(E)** Colony formation assay was performed to examine the colony formation ability of SCC25 cells. Scale bars, 100 μm. One-way ANOVA.** (F)** Western blot results of BCL-2, Bax, Caspase3 and cleaved Caspase3 in co-transfection HNSCC cells. Independent experiments (*in vitro*) were performed in triplicate.** (G)** Live/dead viability assay was conducted to examine apoptosis after co-transfection. Scale bars, 100 μm. One-way ANOVA. Data are presented as mean±SD. *, *P* < 0.05, **, *P* < 0.01, ***, *P* < 0.001, ****, *P* < 0.0001.

To explore whether STAT1 and IRF9 participate in the FAT1-mediated phenotypic transition of HNSCC, we compared eight treatment groups: the empty carrier group, the Lv-STAT1 group (STAT1 overexpression), the Lv-IRF9 group (IRF9 overexpression), the Lv-STAT1+Lv-IRF9 group (STAT1 was cotransfected with IRF9), the Si FAT1 group (siRNA was used to interfere with the expression of FAT1), the Lv-STAT1 and Si FAT1 group, the Lv-IRF9 and Si FAT1 group, and the Lv-STAT1+Lv-IRF9 and Si FAT1 group. The results revealed that the overexpression of STAT1 or IRF9 alone inhibited the stemness of cancer cells and that the co-overexpression of STAT1 and IRF9 strongly suppressed cancer stemness (Fig. S5A-C). Moreover, the overexpression of either STAT1 or IRF9 partially alleviated the enhanced CSC phenotype caused by the knockdown of FAT1, whereas the simultaneous overexpression of STAT1 and IRF9 maximally blocked the effect caused by FAT1 knockdown (Fig. 5A-E and Fig. S5D, S6A). Consistent with these findings, the overexpression of STAT1 and IRF9 inhibited the increased antiapoptotic ability caused by FAT1 knockdown. In addition, the Lv-STAT1 and Si FAT1 group, the Lv-IRF9 and Si FAT1 group, and the Lv-STAT1+Lv-IRF9 and Si FAT1 group exhibited lower expression of BCL-2 and Caspase3 and higher expression of Bax and cleaved Caspase3 than did the Si FAT1 group (Fig. 5F, G and Fig. S6B, C). Taken together, our findings revealed that the STAT1/IRF9 axis participated in the FAT1-mediated phenotypic transition of HNSCC and that the overexpression of STAT1 and IRF9 alleviated FAT1 knockdown-driven CSC properties.

### FAT1 knockdown promotes tumor growth *in vivo*

**Figure 6:**
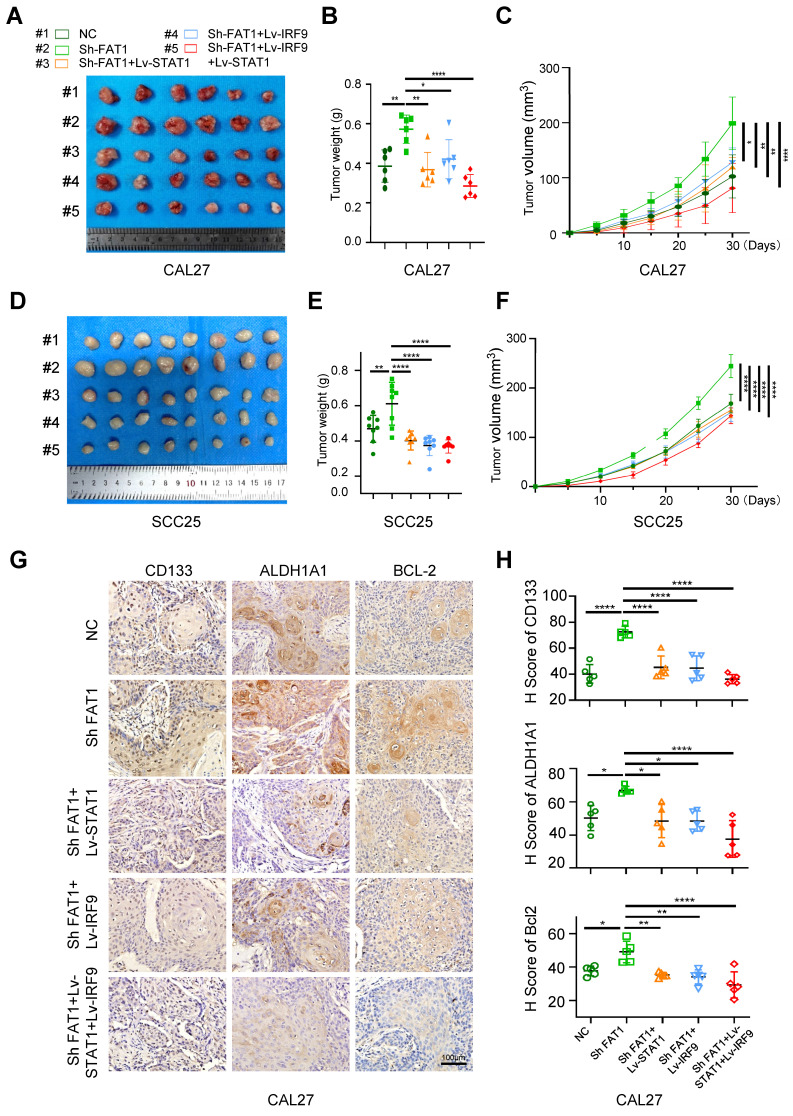
** FAT1 knockdown promotes tumor growth *in vivo*. (A)** Image of xenograft tumors of CAL27 in five groups, grouped as follows: the empty carrier group, the ShFAT1 group (siRNA was used to interfere with the expression of FAT1), the Lv-STAT1 and ShFAT1 group, the Lv-IRF9 and ShFAT1 group, and the Lv-STAT1+Lv-IRF9 and ShFAT1 group. **(B, C)** The volume and weight of the tumor of the subcutaneous tumorigenic model (CAL27). One-way ANOVA.** (D)** Image of xenograft tumors of SCC25 in five groups described in Figure 6A. **(E, F)** The volume and weight of the tumor of the subcutaneous tumorigenic model (SCC25). One-way ANOVA.** (G)** Representative IHC images of CD133, ALDH1A1, and BCL-2 in five groups in xenograft tumors (CAL27). Scale bars, 100 μm. **(H)** Relative IHC score of CD133, ALDH1A1, and BCL-2. One-way ANOVA. Independent experiments (*in vitro*) were performed in triplicate. Data are presented as mean±SD. *, *P* < 0.05, **, *P* < 0.01, ***, *P* < 0.001, ****, *P* < 0.0001.

To investigate the impact of FAT1 on the growth of HNSCC, we constructed a subcutaneous tumor model in nude mice. Five groups of SCC25 and CAL27 cells were prepared *in vitro* separately, including the control and Sh FAT1 (SCC25 and CAL27 cells were transfected with the lentivirus-Si FAT1#3) group (Fig. S6D), the Lv-STAT1 and Sh FAT1 group, the Lv-IRF9 and Sh FAT1 group, and the Lv-STAT1+Lv-IRF9 and Sh FAT1 group. In terms of tumor weight and size, those in the Sh FAT1 group were the largest among all the groups, whereas those in the Lv-STAT1+Lv-IRF9 and Sh FAT1 groups were the smallest; there was no significant difference among the remaining three groups (Fig. 6A-F). The IHC results of the CAL27 cells suggested that the increased expression of CSC-related proteins or decreased expression of apoptosis-related proteins caused by FAT1 knockdown could be eliminated by the overexpression of STAT1/IRF9 (Fig. 6G, H). The TUNEL assay results of the CAL27 cells demonstrated that the overexpression of STAT1/IRF9 counteracted the increased antiapoptotic ability caused by FAT1 inhibition (Fig. S6E). Overall, the inhibition of FAT1 promoted tumor growth *in vivo*, whereas the overexpression of STAT1/IRF9 inhibited the tumor-promoting effect induced by FAT1 knockdown.

## Discussion

**Figure 7:**
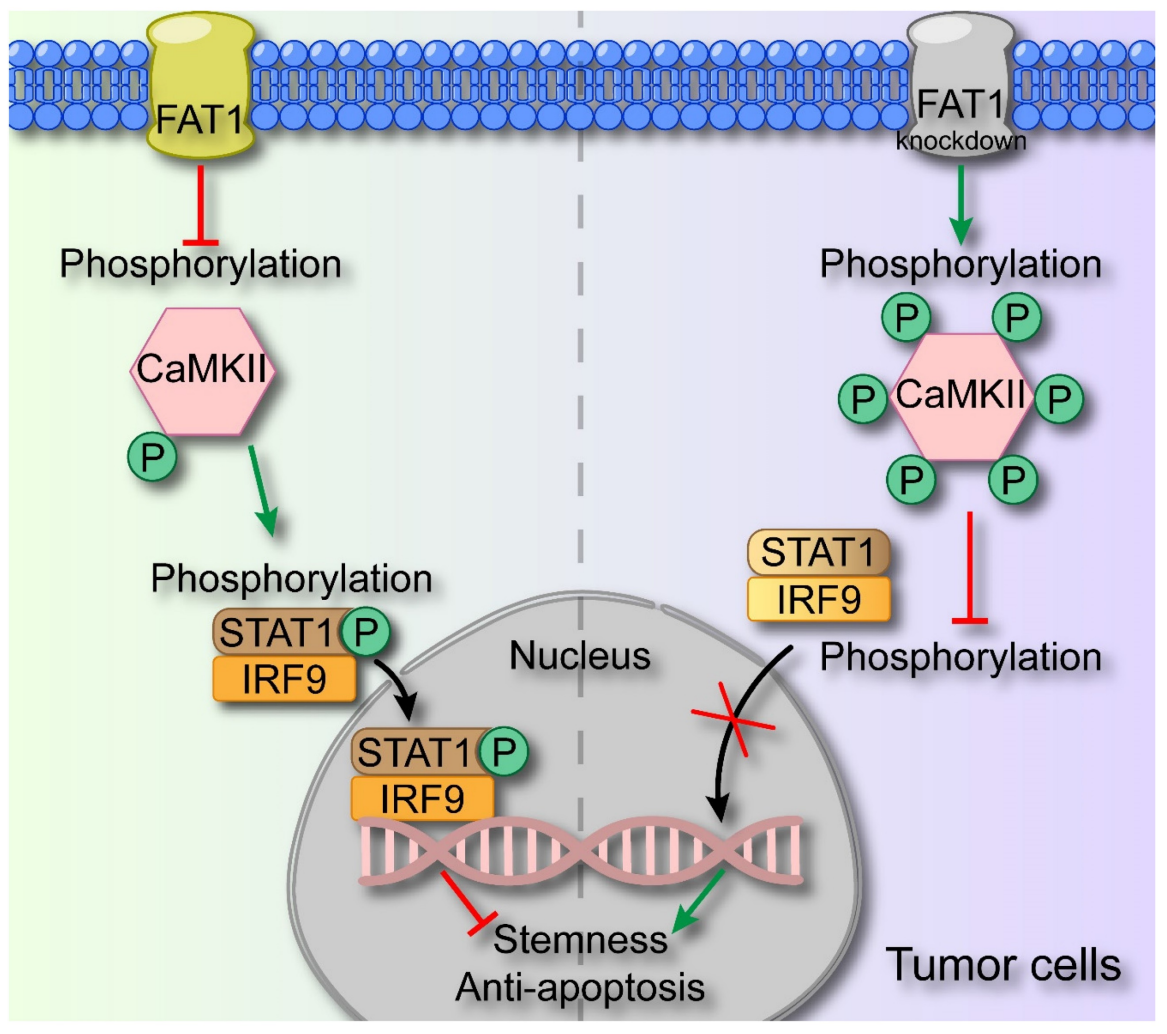
** Schematic diagram of FAT1 functions as a tumor-suppressor in HNSCC.** FAT1 enhances the phosphorylation of CaMKII, which then impedes the formation of the p-STAT1/IRF9 complex. The p-STAT1/IRF9 complex transports to the nucleus and activates type Ⅰ interferon pathway, exerting the cancer-suppressive role. Reduced expression of FAT1 results in the phosphorylation of CaMKII and curbs the formation of p-STAT1/IRF9 complex, leading to enhanced CSC properties of HNSCC.

In this study, we demonstrated that FAT1 suppressed the malignant phenotype of HNSCC through activating the type I interferon signaling pathway. Specifically, we revealed that FAT1 can phosphorylate CaMKII and further impede the formation of the p-STAT1/IRF9 complex, thereby exerting its role in suppressing cancer (Fig. 7). On the basis of our findings, we posit that *FAT1* holds significant promise as a viable target for the treatment of HNSCC.

Recently, *FAT1* mutations have been identified in various cancer types, with its mutation rate in HNSCC second only to that of *TP53*^5^, ^6^, ^22^. In the present study, we found that FAT1 was expressed at low levels in HNSCC tissues/cells and that patients with high FAT1 expression had a better prognosis, suggesting that FAT1 might play a tumor-suppressor role in HNSCC, which is consistent with the findings of previous studies^8^, ^23^-^26^. The CSC theory proposes that tumors consist of a heterogeneous population of cells, and the hierarchy of cells within tumors is maintained by a small group of cells called CSCs, which have continuous self-renewal ability and are strongly resistant to chemoradiotherapy^27^-^30^. Recently, Li *et al.* reported that loss of FAT1 activated the Hippo signaling pathway, which is closely related to the pluripotency of CSCs^31^. Zhai *et al.* revealed that FAT1 inhibited the stemness and ABCC3-related cisplatin resistance of ESCC cells via the Wnt/β-catenin signaling pathway^32^. Hence, we wondered whether FAT1 exerts its cancer-suppressive activity via the modulation of the CSC properties of HNSCC. We interfered with the expression of FAT1 in CAL27 and SCC25 cells via siRNAs and demonstrated that inhibition of FAT1 endowed HNSCC cells with increased CSC properties and a decreased apoptosis rate.

By means of transcriptomic sequencing, we found that FAT1 knockdown affected the type I interferon signaling pathway, which has been recognized to act as a tumor suppressor^33^, ^34^. Here, we noted that treatment with IFN-α resulted in decreased CSC properties and increased the apoptosis rate of tumor cells, indicating that activation of the interferon pathway suppressed the malignant phenotype of tumors. Zhang *et al.* reported that IFN-α strengthened the therapeutic efficacy of EGFR-targeted therapies by augmenting RIG-I, and the m6A demethylase ALKBH5 could negatively regulate RIG-I expression and IFN-α production through the IKKε/TBK1/IRF3 pathway in HNSCC^35^, ^36^. However, few efforts have been directed toward investigating the interplay between FAT1 and the interferon pathway. STAT1 and IRF9 serve as pivotal transcription factors within the type I interferon signaling pathway, and the formation of the p-STAT1/IRF9 complex represents the activation of this pathway^37^. The transcriptomic sequencing results revealed that the expression of STAT1 and IRF9 was significantly upregulated after FAT1 knockdown, indicating the involvement of the interferon pathway. However, we found that IRF3, which triggers the secretion of type I IFNs, remained largely unaffected upon FAT1 knockdown^13^, and the secretion of type I IFNs was only slightly greater than that in the control group. We found that interfering with the expression of IFN-α or IFN-β via siRNA treatment had a negligible effect on FAT1-mediated alterations in the interferon pathway. Therefore, we hypothesize that FAT1 activates the type I interferon pathway independent of IFN-α and IFN-β secretion. Pastushenko *et al.* reported that FAT1 regulated the phosphorylation of CaMKII, which then directly or indirectly phosphorylated YES and SRC^8^. As an enzyme, CaMKII can react with a substrate to promote its phosphorylation, or it can prevent other kinases from binding to the substrate, thereby exerting competitive inhibition to prevent substrate phosphorylation^38^. Here, we found that FAT1 directly regulated the type I interferon pathway by inhibiting the phosphorylation of CaMKII, which subsequently phosphorylated STAT1 and promoted the formation of the p-STAT1/IRF9 complex. Knocking down CaMKII significantly blocked the effect of FAT1 on the type I interferon pathway. Additionally, the overexpression of STAT1 and IRF9 rescued the inactivation of the type I interferon pathway following FAT1 knockdown.

In the present study, we revealed a previously unreported mechanism through which FAT1 exerts its tumor-suppressor effect by impeding the type I interferon pathway. This novel insight highlights the role of FAT1 in the field of HNSCC. However, our focus was confined to the role of FAT1 in the malignant attributes of tumor cells, omitting an investigation into the underlying causes of *FAT1* mutation and its contribution to the transformation of normal cells into malignant tumor cells. Although we performed *in vivo* replicated experiments using both cell lines, the subcutaneous xenograft model is not an ideal model, and its lack of in situ tumour microenvironment (e.g. specific blood flow and tissue characteristics) and metastatic properties does not fully reflect the reality *in vivo*. In addition, the type I interferon pathway is intricately intertwined with immunity, yet our exploration did not extend to investigating the influence of FAT1 on tumor immunity.

In summary, our findings indicate that FAT1 knockdown prevents the activation of the type I interferon pathway through a noncanonical CaMKII-dependent pathway. As a result, directing therapeutic attention toward FAT1 holds significant promise as a potentially effective strategy for addressing HNSCC.

## Materials and Methods

### Cell lines and culture

The human HNSCC cell lines HN4, SCC9, SCC25, HaCaT, and CAL27 were purchased from the Chinese Academy of Science (Shanghai, China). Human immortalized oral epithelial cells (HIOECs) were a gift from Professor Chengzhang Li. Both SCC9 and SCC25 cells were cultured in DMEM/F12, and HN4, HaCaT and CAL27 cells were cultivated in DMEM/high glucose. HIOECs were cultured in KGM™ Gold Keratinocyte Cell Basal Medium (Lonza, Switzerland) supplemented with associated growth factors. All the cells were maintained in medium supplemented with 10% fetal bovine serum (Gibco, USA) at 37 °C in a humidified incubator with 5% CO2. All the cells were tested and confirmed to be free of mycoplasma and chlamydia contamination.

### Specimen collection

HNSCC tumor tissues and normal oral mucosa samples were collected at the Hospital of Stomatology, Wuhan University. Our experiments were approved by the Ethics Committee of the School and Hospital of Stomatology, Wuhan University (2022A05). Written informed consent was obtained from all patients.

### Coimmunoprecipitation (Co-IP)

CAL27 and SCC25 cells transfected with Si FAT1 or control siRNAs were lysed for 0.5 h on ice after transfection in the presence of CaMKII knockdown. Following lysate centrifugation, the supernatants of the samples were mixed well with primary antibody/IgG at 4 °C overnight. The next day, the mixture was mixed with protein magnetic A/G beads (Bimake, B23202, China) at room temperature for 1 hour, heated at 95 °C for 10 min after adding 5×SDS‒PAGE buffer, and then analyzed by western blotting.

### Sphere formation assay and colony formation assay

For the sphere formation assay, tumor cells were digested with pancreatic enzymes to form a single-cell suspension. They were then cultured in ultralow-attachment plates at a density of 10,000 cells/well in serum-free DMEM/F12 supplemented with 2% B27 (Thermo Fisher Scientific, USA), human recombinant EGF (20 ng/ml, PeproTech, Bubendorf, Switzerland), and human recombinant basic fibroblast growth factor (10 ng/mL, PeproTech, Bubendorf, Switzerland). Images were taken to capture the shape and size of the spheres after 10~14 days, after which the number of spheres per well was counted.

Like in the sphere formation assay, the isolated cells were plated in a 12-well plate with full medium, fixed with 4% paraformaldehyde, and stained with crystal violet. Finally, a photo was taken, and the number of colonies with more than 50 cells was counted.

### *In vitro* limiting dilution assay

Initially, a single-cell suspension was obtained. Four doses (1000, 100, 10, and 1) of CAL27 and SCC25 cells were subsequently plated in ultralow-attachment 96-well plates. The frequency of CSCs was determined via the extreme limiting dilution analysis (ELDA) web tool. Specific experimental steps were followed according to the protocol provided in Bioprotocol^17^.

### Immunohistochemistry

For immunohistochemistry (IHC), tumor tissue samples excised from BALB/c nude mice were sectioned to 5μm thickness, dewaxed with xylene, dehydrated with alcohol, subjected to microwave-repaired antigen, and blocked with 5% BSA at 37 °C for 1 h. The samples were incubated with the primary antibody overnight at 4 °C, followed by incubation with the secondary antibody for 1 h at room temperature. Then, color development was performed via DAB, the samples were allowed to dry, the samples were sealed, and photos were captured via a microscope^39^. Subsequently, the images were converted to RGB format using ImageJ software, and the black-and-white images were adjusted to enhance the contrast and make the target area clearer. Next, the positive areas were completely covered by red color by setting a threshold and finally the optical density values were measured to quantify the results. Subsequently, the GraphPad Prism 8 software was utilised for quantitative and statistical analysis. Patient tissue-specific information used was in Supplementary Excel file 3. And characterization of HNSC patients and correlation between FAT1 expression and clinicopathological variables were shown in Table S3.

### Mouse xenografts

Five-week-old female BALB/c nude mice were purchased from Beijing Vital River Laboratory Animal Technology Co., Ltd. (Beijing, China) and were housed under standard laboratory conditions. The xenograft studies were approved by the Ethical Committee on Animal Experiments of the Animal Care Committee of Wuhan University (S0792203066). Female BALB/c nude mice were randomly divided into five groups after approximately 1×10^6^ CAL27 and SCC25 cells transfected with lentivirus were injected subcutaneously into the right dorsal region of mice. Tumors were harvested upon reaching the end point of the trial.

### Statistical analysis

Statistical analysis was performed with GraphPad Prism 8 software. Comparisons between groups were analyzed via two-tailed unpaired Student's t test (two groups) or one-way ANOVA followed by Dunnett's multiple comparison test (more than two groups). The results are expressed as the means ± SDs and were considered statistically significant when* p* < 0.05.

## Supplementary Material

Supplementary materials and methods, and tables.

Supplementary excel file 1.

Supplementary excel file 2.

Supplementary excel file 3.

Supplementary excel file 4.

Supplementary excel file 5.

Supplementary excel file 6.
