# Supplementary material for: FAT1 knockdown enhances the CSC properties of HNSCC through p-CaMKII-mediated inactivation of the IFN pathway
Source: Int J Biol Sci. 2025 Jan 1;21(2):671–84. doi: 10.7150/ijbs.95723 (PMC11705627; doi:10.7150/ijbs.95723)
Supplement: Supplementary file 1 — Supplementary materials and methods, and tables. [file ijbsv21p0671s1.pdf]

# FAT1 knockdown enhances the CSC properties of HNSCC through p-CaMKII-mediated inactivation of the IFN pathway

Jingjing Wang [1,#], Yang Chen [1,3,#], Yunqing Sun [1], Hanzhe Liu[1], Ruixue Du [1], Xuewen Wang [1], Zhe Shao[1,2], Ke Liu[1,2,*], Zhengjun Shang[1,2,*]

[1] State Key Laboratory of Oral & Maxillofacial Reconstruction and Regeneration, Key Laboratory of Oral Biomedicine Ministry of Education, Hubei Key Laboratory of Stomatology, School & Hospital of Stomatology, Wuhan University.

[2] Department of Oral and Maxillofacial-Head and Neck Oncology, School & Hospital of Stomatology, Wuhan University, Wuhan 430079, China.

[3] Department of Oral and Maxillofacial Surgery, School & Hospital of Stomatology, Wuhan University, Wuhan 430079, China.

**# Jingjing Wang and Yang Chen contributed equally to this study.**

***Corresponding authors:** Zhengjun Shang and Ke Liu, State Key Laboratory of Oral & Maxillofacial Reconstruction and Regeneration, Key Laboratory of Oral Biomedicine Ministry of Education, Hubei Key Laboratory of Stomatology, School & Hospital of Stomatology, Wuhan University, Wuhan 430079, China. Fax: +86-27-87873260. E-mail: shangzhengjun@whu.edu.cn (Z, Shang), liuke.1999@whu.edu.cn (K, Liu).

# Materials and Methods

## 2.1 Cell lines and culture

IFN-α (Interferons) were purchased from MedChemExpress (HY-P7022, USA) and dissolved with deionized water. KN93 was purchased from MedChemExpress (HY-15465B, USA) and dissolved in sterile distilled water. CD133 (20874-1-AP), CD44 (10366-1-AP), ALDH1A1 (12129-1-AP), OCT4 (10366-1-AP), NANOG (10366-1-AP), BMI1 (12129-1-AP), β-acting (10366-1-AP), Bcl-2 (12789-1-AP), Caspase3 (19677-1-AP), Bax (50599-2-Ig), STAT1 (10144-2-AP), P-STAT1 (28977-1-AP), STAT2 (16674-1-AP), P-STAT2 (39612), IRF9 (14617-1-AP), HSP90 (60318-1-Ig), IFN-α (18013-1-AP), IFN-β (27506-1-AP) antibodies mentioned above were purchased from Proteintech (Wuhan, China). Two FAT1 antibodies were obtained from Abcam, the one used for western blot (ab190242, USA), and the other one used for immunohistochemistry (ab198892, USA). p-CaMKII antibodies were obtained from Cell Signaling Technology (12716, USA).

## 2.2 CCK-8 and EdU assay

Seeding $5\times10^3$ CAL27 and SCC25 cells per well in 96-well plates, and then incubating them with Cell Counting Kit-8 (CCK-8) solution (Beyotime, China) for 2 h after cells grew up to 40%~50%. Then, follow the manufacturer's instructions to examine the proliferation ability of the cells.

Initially, $2\times10^5$ cells were planted in the 12-well plates with cell climbing glasses, using the EdU Cell Proliferation Kit (Beyotime, China) as instructed after 24~48 h, washing cells with PBS for three times, fixed with formaldehyde and staining with the

kits, finally obtained the photo through the fluorescence microscope bought from Keyence (Biozero BZ-8000, Japan).

## 2.3 Wound healing assay and Matrigel invasion assay

For the wound healing assay, the full medium was replaced with a serum-free medium upon CAL27 and SCC25 cells reached 80%~90% confluence in 6-well plates. At the same time, two scratches of nearly the same width were created in each well. Taking a photo at the 0 and 48 h at the same point, and using ImageJ to measure the scratch areas.

Regarding the matrigel invasion assay, transwell chamber plates with 8μm filter wells were obtained from Corning (Tewksbury, USA) and matrigel was bought from BD (Biosciences, USA) respectively. Cells suspended in FBS-free medium were added to the upper chamber, while 500μL full medium was loaded in the lower chamber. After 48 h, wash and wiped out the cells in the upper chamber, and stained them with crystal violet.

## 2.4 RNA isolation and real-time quantitative PCR

Total RNA was extracted from cells by using RNAiso Plus (Takara, Japan) with enzyme-free throughout. A total of 1μg of RNA was utilized for reverse transcription using the HiScript RT reagent kit (Vazyme Biotech, China), resulting in cDNA synthesis. The levels of mRNA were quantified using ChamQ SYBR qPCR Master Mix (Vazyme Biotech, China), with GAPDH serving as an internal control. The primer sequences used were mentioned in Supplementary table 2 (Supporting Information). The expression of mRNA was quantitated through the $2^{-\Delta\Delta Ct}$ method and trials were carried out in triplicate. And the results of RT-PCR are detailed in Supplementary Excel

file 1.

## 2.5 RNA interference and lentiviral transfection

For RNA interference, three siRNAs targeting FAT1, IFN-α, IFN-β, CaMKII and control RNAs were bought from Genepharm (Suzhou, China), the siRNA sequences used are listed in Supplementary table S1. The siRNA-Lipo3000 mixture was added to the cell dishes with FBS-free medium for 48~72 h, once the cells reached 70%~90% confluence.

For lentivirus transfection, CAL27 and SCC25 cells were seeded in 12-well plates at 30%~40% confluence and incubated overnight, then transfected with an empty carrier lentivirus or a STAT1/IRF9 overexpression lentivirus (GeneChem, China) for 24 h. Subsequently, the cells were selected with puromycin (6 mg/L) for 24 h.

## 2.6 Immunofluorescence

For cell climbing glasses, wash them with PBS twice, fix with 4% paraformaldehyde, permeabilize with 0.3% Triton X-100, and block with 5% BSA at 37°C for 1 h. After an 8 h incubation with the primary antibody at 4°C, choose a proper fluorescent secondary antibody, incubate samples for 1 h at room temperature, mount them using an anti-fluorescent quenching agent, and capture images using a fluorescent microscope.

## 2.7 Western blot

Discarding medium with PBS washing out three times, then harvesting protein using RIPA supplemented with a cocktail of protease inhibitors and phosphatase inhibitors. The BCA Protein Assay Kit (Beyotime, China) was used to determine the protein concentration. The lysates-SDS mixture was heated at 95°C for 10 min. 20~40μg

proteins were separated on 6%~12% SDS-PAGE gels and electrophoresed at a 200mA constant current for 150 min to polyvinylidene fluoride membranes. Incubate with primary antibodies at 4°C overnight after blocking with Quick Block (Beyotime, China) for 15 min, followed by a horseradish peroxidase–conjugated mouse or rabbit secondary antibody for 1 h at 37°C. Washed the membranes three times with TBST and visualized the blot by the Odyssey System (Li-Cor Biosciences). The densitometric data for all WB images are available at Supplementary Excel file 5.

## 2.8 Determination of IFNs concentration

Firstly, SCC25 and CAL27 were seeded into 6-well plates at a proper density. Exchanging full medium with the serum-free medium when the cell confluence reached 40%~50%, and then harvested the upper clear after 48 h. The upper clear was centrifuged at 1000 rpm for 5 min at 4 °C, discarding the sedimentation before determining these samples.

Human IFN-α ELISA Kit was brought from Neobioscience (EHC144a, China), and the human IFN-β ELISA Kit was acquired from Neobioscience (EHC026b, China), next followed by the product instructions to measure the concentration of IFN-α and IFN-β.

## 2.9 Annexin V-Pacific blue/PI dual staining and Live/dead viability assay

First of all, harvest almost 5~10×$10^5$ cells and wash them twice with PBS. Next, follow the Annexin V-Pacific blue/PI dual staining assay protocol (E-CK-A211, Elabscience Biotechnology). The CAL27 and SCC25 cells were sorted using flow cytometry (BD,

Biotechnology).

For the live/dead viability assay, planted $5 \times 10^5$ cells per well in 6-well plates, the live /dead viability assay (Beyotime, China) was employed according to the protocol upon cells reached 90% confluence. Finally, obtain images with a fluorescent microscope.

## 2.10 RNA-sequencing and Bioinformatics analysis

The cells were transfected with Si FAT1 into CAL27 cells. 48 hours after transfection, the supernatant was discarded, washed three times with PBS, and the tumor cells were harvested with a cell scraper and immediately frozen in liquid nitrogen, and then sent to Shenzhen Huada Gene Science and Technology Service Co. for subsequent extraction, preparation of libraries and sequencing analysis. The raw data for RNA-Seq is provided in the Supplementary Excel file 4.

The relationship between FAT1 expression and tumor cell stemness (CSC score) was analysed by the SangerBox platform (http://sangerbox.com/home.html), and the results were shown in Figure 2A, with the CSC score based on the previously published paper Machine Learning Identifies Stemness Features Associated with Oncogenic Dedifferentiation (PMID: 29625051) was determined. Subsequently, correlation analyses were performed based on the expression levels of FAT1 mRNA and CSC scores, and the specific data are also shown in Supplementary Excel file 6.

Table S1. The sequences of siRNAs.

| Oligonucleotides | Sequences |
|---|---|
| SiFAT1-1 | 5’-3’CUAGAGACCAAGCUCUAUTT |
| SiFAT1-1 | 3’-5’AUAGAGCUUGGUCUCUAGGTT |
| SiFAT1-2 | 5’-3’GCAACCGGCUCUCUCUAUATT |
| SiFAT1-2 | 3’-5’ UAUAGAGAGAGCCGGUUGCTT |
| SiFAT1-3 | 5’-3’GGAGCAAGCUGUUUAUCAUTT |
| SiFAT1-3 | 3’-5’ AUGAUAAACAGCUUGCUCCTT |
| SiCAMKⅡ-1 | 5’-3’ AGAGCGATGGTGTGAAGGAAUTT |
| SiCAMKⅡ-1 | 3’-5’ AAATTCCTTCACACCATCGCTTT |
| SiCAMKⅡ-2 | 5’-3’ GGGACACCACTACCTGATCTUTT |
| SiCAMKⅡ-2 | 3’-5’ AAAAGATCAGGTAGTGGTGTCTT |
| SiIFNα-1 | 5’-3’ CCTCTCTTTATCAACAAACTUTT |
| SiIFNα-1 | 3’-5’AAAAGTTTGTTGATAAAGAGATT |
| SiIFNα-2 | 5’-3’ CCTGGATAACAGGAGGACCTUTT |
| SiIFNα-2 | 3’-5’AAAAGGTCCTCCTGTTATCCATT |
| SiIFNβ-1 | 5’-3’ CGCATTGACCATCTATGAGAUTT |
| SiIFNβ-1 | 3’-5’AAATCTCATAGATGGTCAATGTT |
| SiIFNβ-2 | 5’-3’ GAGCTACAACTTGCTTGGATUTT |
| SiIFNβ-2 | 3’-5’AAAATCCAAGCAAGTTGTAGCTT |

Table S2. Primers used in the study.

| Primers | Sequences（5'-3'） |
|---|---|
| CD44 forward primer | CTGCCGCTTTGCAGGTGTA |
| CD44 reverse primer | CATTGTGGGCAAGGTGCTATT |
| CD133 forward primer | AGTCGGAAACTGGCAGATAGC |
| CD133 reverse primer | GGTAGTGTTGTACTGGGCCAAT |
| ALDH1A1 forward primer | CCGTGGCGTACTATGGATGC |
| ALDH1A1 reverse primer | GCAGCAGACGATCTCTTTCGAT |
| GAPDH forward primer | ACAACTTTGGTATCGTGGAAGG |
| GAPDH reverse primer | GCCATCACGCCACAGTTTC |
| Bax forward primer | CATATAACCCCGTCAACGCAG |
| Bax reverse primer | GCAGCCGCCACAAACATAC |
| Bcl-2 forward primer | GTCTTCGCTGCGGAGATCAT |
| Bcl-2 reverse primer | CATTCCGATATACGCTGGGAC |

Table S3. Characterization of HNSC patients and correlation between FAT1 expression and clinicopathological variables.

| Characteristics | No. of patients | FAT1 expression | | P-value |
|---|---|---|---|---|
| | n=520 | Low(n=260) | High(n=260) | |
| Age | | | | |
| ≤60 | 258 | 124 | 134 | 0.0867 |
| ＞60 | 261 | 136 | 125 | |
| missing | 1 | 0 | 1 | |
| Gender | | | | |
| Female | 384 | 198 | 186 | 0.6921 |
| Male | 136 | 62 | 74 | |
| Missing | 0 | 0 | 0 | |
| Clinical stage | | | | |
| I+II | 118 | 56 | 62 | 0.0617 |
| III+IV | 388 | 193 | 195 | |
| missing | 14 | 11 | 3 | |
| Histological grade | | | | |
| G1+G2 | 366 | 182 | 184 | 0.4467 |
| G3+G4 | 132 | 64 | 68 | |
| missing | 22 | 14 | 8 | |
